# Preparation and Functional Properties of Synbiotic Yogurt Fermented with *Lactobacillus brevis* PML1 Derived from a Fermented Cereal-Dairy Product

**DOI:** 10.1155/2021/1057531

**Published:** 2021-08-12

**Authors:** Fereshteh Falah, Alireza Vasiee, Farideh Tabatabaee Yazdi, Behrooz Alizadeh Behbahani

**Affiliations:** ^1^Department of Food Science and Technology, Faculty of Agriculture, Ferdowsi University of Mashhad, Mashhad, Iran; ^2^Department of Food Science and Technology, Faculty of Animal Science and Food Technology, Agricultural Sciences and Natural Resources University of Khuzestan, Mollasani, Iran

## Abstract

Nowadays, production of functional foods has become very essential. Inulin is one of the most functional hydrocolloid compounds used in such products. In the present study, the production of a synbiotic yogurt containing 1, 2.5, and 5% (*w*/*v*) inulin has been investigated. The yogurt was fermented with *Lactobacillus brevis* PML1 derived from Tarkhineh, an Iranian cereal-dairy fermented food. Furthermore, the physicochemical properties, antioxidant activity, sensory attributes, and microbial viability properties were investigated on the 0th, 7th, and 14th days of storage after fermentation. The viable cells of *L. brevis* PML1 reached 10^8^ CFU/g, and the product resisted to simulated digestive juices. Moreover, the synbiotic yogurt impressively increased the production of antimicrobial compounds and had the most profound antimicrobial effect on *S. typhimurium*. The physiochemical properties were in the normal range, and the fat content of the synbiotic yogurt was reduced remarkably. The antioxidant capacity of the fermented yogurt was significantly increased (*p* < 0.05), which was equal to those of DPPH (69.18 ± 1.00%) and BHA (89.16 ± 2.00%). The viability of *L. brevis* PML1 was increased during storage. Sensory analysis showed that there were significant differences in terms of the impressive parameters between the samples and the control (*p* < 0.05). Addition of 2.5% inulin not only improved the physical properties but also retained the viability of the probiotic after 14 days of storage, in addition to the viability of *L. brevis* with a viability count above 6 log CFU/g in the yogurt. Therefore, a novel synbiotic product containing *L*. *brevis* PML1, which can exert the desired properties, can be used as a suitable carrier for the delivery of the probiotic strain, exerting its beneficial health effects.

## 1. Introduction

Yogurt is an excellent source of essential nutrients, so it has become one of the most popular options for producing pragmatic and healthy foods [1]. As yogurt is growing in popularity, manufacturers are continually looking for value-added ingredients such as probiotics to produce functional yogurt with more beneficial properties. In this study, the yogurt samples, including the control and fermented by *L. brevis* PML1 as a probiotic strain, containing different inulin concentrations were produced [2]. In recent decades, the tendency towards the use of natural or processed functional foods containing bioactive compounds has been expanded, which has become a health benefit and provided the basis for further research. Nowadays, customers' demand for healthful dairy stuffs such as probiotic and synbiotic products has improved [3]. Yogurt or yoghurt is known as an appreciated dairy product available in various textures, fat contents, and flavors. Standard yogurt is a kind of fermented milk product with a distinct texture and a soft agreeable taste, which results from fermentation with *Lactobacillus delbrueckii* ssp. *bulgaricus* and *Streptococcus thermophilus*. Bioyogurt or synbiotic yogurt is fermented with probiotic bacteria such as *Bifidobacterium* and *Lactobacillus* strains declared to have various health benefits and should remain alive at sufficient percentages. Yogurt is an excellent nutritional transporter for the efficient transition of useful microorganisms into the human body [4, 5].

Probiotics are defined as live microorganisms which when consumed in a sufficient amount could confer a health benefit. Probiotics must be able to survive at all stages of food storage and must not change its physicochemical or sensory properties. LAB are Gram-positive aerotolerant homo-fermentative bacteria and a L-(+)-lactic acid producer. To improve the functionality of food matrices, many dairy foodstuffs are being improved with indigestible oligosaccharides, like inulin, as prebiotic ingredients [6]. This oligosaccharide is optionally fermented in the colon by microbiota and can consequently assign countless natural benefits. Probiotics have favorable effects on the host's health such as cancer control, cholesterol reduction, and modulation of the immune system, facilitating and increasing the mineral absorption and human immunity. By using proper substrates of probiotics, their viability can be improved in various products. When a product contains both probiotics and prebiotics, it can be called synbiotic. The most commonly used probiotic strains are *Lactobacillus* spp. including *Lactobacillus acidophilus*, *Lactobacillus rhamnosus*, *Lactobacillus plantarum*, and *Lactobacillus brevis* [7].

Tarkhineh is a fermented cereal-dairy product. Because of its high nutritional profits, it could be regarded as a good source of proteins, amino acids, fatty acids, and minerals. It is usually produced with buttermilk and turnip [8]. It is subsequently fermented with the microbiota of doogh at room temperature. The probiotic potential of *L. brevis* PML1 isolated from Tarkhineh has been investigated by Falah et al. [9]. The results indicated that the subjected strain had a high potential for survival in acidic conditions and bile salts. The percentages of hydrophobicity and adhesion were 18.33% and 13.8%, respectively. Additionally, *L. brevis* PML1 showed a high ability to prevent the pathogenicity of adhesion (35.5%).

Prebiotics are indigestible biopolymers which are able to improve the activity of microbiota and their viability, thus producing beneficial effects on consumers' health. Inulin has widely been reported to be a prebiotic improving the viability of colonic microbiota [10]. The short-chain fraction is much more soluble and sweeter than the long-chain inulin. It can also improve mouth-feel and impression, because of its textural properties. Long-chain inulin is less soluble with higher viscosity, which can act as a texture modifier [11]. The purpose of this study was to evaluate the physical, chemical, and microbial effects of synbiotic yogurt containing a native probiotic strain (*L. brevis* PML1) and inulin simultaneously. Analysis and comparison of the different yogurt samples and different inulin concentrations were also evaluated during refrigerated storage at three levels of storage time (0, 7, and 14 days).

## 2. Materials and Methods

### 2.1. Bacterial Growth

*L. brevis* PML1, as a confirmed probiotic candidate, was chosen as the adjunct culture for yogurt production in this study. This strain was cultured on De Man, Rogosa, and Sharpe (MRS) at 37°C for 24 h. *Staphylococcus aureus* ATCC 25923, *Listeria innocua* ATCC 33090, *Escherichia coli* ATCC 25922, and *Salmonella typhi* ATCC 6539 were procured from microbial collection, Department of Food Science and Technology, Faculty of Agriculture, Ferdowsi University of Mashhad, Iran. These selected pathogens were inoculated into Muller Hinton Broth (Quelab, Canada) and incubated at 37°C for 24 h [12].

### 2.2. Production of Synbiotic Yogurt

In order to produce the probiotic yogurt, fresh cow milk containing low-chain inulin at three concentrations (0, 2.5, and 5%, *w*/*v*) was heated at 85°C for 20 min. Then, it was cooled to 45°C and inoculated with traditional starter culture (*Lactobacillus delbrueckii* ssp. *bulgaricus* and *Streptococcus thermophilus)* as well as *L. brevis* PML1 as the probiotic culture (LAB, 10^6^ CFU/mL) and incubated at 37°C for 18 h. The samples were stored at low temperature for 0, 7, and 14 days [13].

### 2.3. Physical Properties

pH values were determined using a pH meter (Metrohm, Switzerland). The acidity of the yogurt samples was measured according to Iranian National Standard No. 2852 on the 0th, 7th, and 14th days of storage [14] .

### 2.4. Determination of Water Holding Capacity (WHC)

For measuring the WHC of the samples, 5 g of yogurt was centrifuged at 4000 rpm for 30 min at 10°C. Afterwards, WHC was calculated by the following formula:

(1) $$\begin{array}{c} \text{WHC}=1–\left(\frac{W_{t}}{W_{i}}\right)*100, \end{array}$$

where *W*_*t*_ is the supernatant weight (g) and *W*_*i*_ is the primary weight (g) of the sample [15].

### 2.5. Syneresis Evaluation

The syneresis of the samples was measured on days 0, 7, and 14 of storage. 40 g of the yogurt sample was centrifuged at 5000 rpm for 10 min at 4°C. The supernatant was discarded, and the pellet was weighed. The syneresis of the yogurt samples was expressed as the ratio between the measured weight of the final yogurt and the initial weight of the yogurt [13].

### 2.6. Viscosity Measurements

The apparent viscosity of the yogurt samples was measured at 24°C 24 h after preparation. The output data were recorded and analyzed by Bohlin model visco 88–viscometer (England) [16].

### 2.7. Texture Profile Analysis (TPA)

TPA measurements were carried out using the “Universal Texture Analyser” (QTS Texture Analyser; CNS, Farnell, the UK) connected to a computer programed with the Texture ProTM texture analysis software. A flat-ended rod probe (25 mm in diameter) was attached to a 2 kg compression load while the target value was set at 10 mm with a speed of 0.5 cm/min. The samples (50 g) were placed on glass Petri dishes (13 cm in diameter and 1.5 cm deep). The probe was set to penetrate into the samples to a depth of 0.8 cm. TPA resulted in the calculation of instrumental hardness (the peak force measured during the first compression cycle, F2), instrumental adhesiveness (the negative force area for the first bite, representing the work necessary to pull the compression probe away from the sample, based A3), and instrumental gumminess (the product of hardness and cohesiveness, F2 ∗ A2/A1), where A1 and A2 are the areas under the compression curves of the first and the second bites, respectively [17].

### 2.8. Sensory Evaluation

Expert sensory panelists participated in this study. The panel of the assessors comprised 8 males and 7 females aged between 25 and 38 years, selected from the students of Ferdowsi University of Mashhad. They were asked to evaluate the sensory attributes of the samples. All the panelists were seated in separate booths, and the samples were presented under a red-green light to avoid visual bias. The samples were assessed for odor, taste, texture, and overall acceptability on a 5-point hedonic scale [18].

### 2.9. Chemical Tests

Total solids, protein, and fat contents were evaluated using AOAC methods. The weight of the residue obtained after the moisture content determination was expressed as the percentage of the total solids using the formula below [19]:

(2) $$\begin{array}{c} \text{Total solids }\left(\%\right)=\frac{\left(\text{Weight of dish}+\text{Dry yoghurt}\right)-\text{Weight of dish}}{\text{Weight of the sample}}\times 100. \end{array}$$

The levels of total nitrogen (TN), nitrogen soluble at pH 4.6 (SN), and nonprotein nitrogen (NPN) were determined, as well as in the mixes, before and after heating by the Kjeldahl method. All the measurements were carried out in duplicate. A multiplication factor of 6.38 was used to convert nitrogen to protein. The nonprotein fraction (NP), expressed as protein equivalent, was calculated as NPN × 6.38. The true protein fraction (TP) was calculated as (TN − NPN) × 6.38. The fraction of the protein soluble at pH 4.6 (SP) was calculated as (SN − NPN) × 6.38. The fraction of the protein insoluble at pH 4.6 (IP) was calculated as (TN − SN) × 6.38. The fraction of the protein soluble at pH 4.6 was determined in the mixes before and after heating (SP1 and SP2, respectively). The extent of protein denaturation (*D*) occurring in the mixes during heating was calculated as described below [20]:

(3) $$\begin{array}{c} D\;\left(\%\right)=\frac{\text{SP}1-\text{SP}2}{\text{SP}1}\times 100. \end{array}$$

The fat content was determined by the modified Mojonnier ether extraction method (AOAC, 1995). The extracted fat was dried to a constant weight and expressed as the percentage of fat by weight [19].

### 2.10. Color Analysis

The color parameters, namely, *L*∗ (lightness darkness), *a*∗ (red-green axis), and *b*∗ (yellow-blue axis), of the yogurt samples were determined at 8°C using standard cuvettes for the assessment of liquids. High-resolution images of the samples were captured by a digital camera. The images were then analyzed by Adobe Photoshop for Windows® ver. 8 [21].

### 2.11. Antioxidant Activity

#### 2.11.1. 2,2-Diphenyl-1-Picrylhydrazyl (DPPH) Assay

37.5 *μ*L of each yogurt sample was mixed with 2 mL of the DPPH methanol solution. The samples were incubated in the dark for 20 min, and their absorbance values as well as that of the reference (methanol) were measured at 517 nm [22]. The following equation was used to calculate the DPPH radical scavenging activity:

(4) $$\begin{array}{c} \text{Scavenging activity }\left(\%\right)=\left(\frac{\left[A_{\text{control}}–A_{\text{sample}}\right]}{A_{\text{control}}}\;\right)\times 100, \end{array}$$

where *A*_*control*_ denotes the absorbance value of the control reaction and *A*_*sample*_ represents the absorbance value of the sample. BHA and ascorbic acid were used as positive controls [23].

#### 2.11.2. 2,20-Azino-Bis-3-Ethylbenzothiazoline-6-Sulfonic Acid (ABTS) Assay

ABTS radical cations were investigated by reacting ABTS in reagent. The mixture was shaken and left in the dark for 12 h before use. The yogurt sample was mixed with the ABTS solution and subsequently placed in a dark chamber for 10 min. The absorbance value was recorded at 734 nm, and the scavenging activity (%) was calculated as below [24]:

(5) $$\begin{array}{c} \text{Inhibition}\%=\left(\frac{\left[A_{\text{control}}–A_{\text{sample}}\right]}{A_{\text{control}}}\;\right)\times 100. \end{array}$$

### 2.12. Microbial Analysis

One g of the yogurt was mixed with 9 mL of normal saline (a solution of 0.9% (*w*/*v*( NaCl (Merck, Darmstadt, Germany)) and diluted to a concentration of 10^−6^ and 10^−7^. Then, 1 mL of each dilution was repeated in 2 plates containing MRS Agar (Merck, Darmstadt, Germany) [24]. The bacteria were counted by the pour plate technique [25].

### 2.13. *In Vitro* Growth Control of Selected Pathogens by Synbiotic Yogurt

The pathogenic strains were cultured under the mentioned conditions and subjected to centrifugation at 8000 g for 10 min at 4°C. The supernatant was subsequently discarded, and the pellets were washed with cold sterile PBS (pH 7.0) and then dissolved in the same buffer so that the final number of bacteria would be 10^6^ CFU/mL. Each prepared pathogen suspension was added (10%, *w*/*v*) to 10 g of the synbiotic yogurt samples with different inulin concentrations.

Process of the pathogenic bacteria, the yogurt samples were kept at 20°C, and the microbial analysis was performed on the days 0, 7, and 14 by the pour plate method onto MHA. The plates were incubated at 48°C for 2 days, and finally, the reduction (%) in the initial number of the pathogenic strains was calculated [26].

### 2.14. Statistical Analysis

The tests were triplicated, and the results were expressed as mean ± standard deviation (SD). The results were analyzed using analysis of variance (ANOVA) followed by Fisher's exact test with *α* = 0.05. For+ this analysis, the software of SPSS v. 18.0 was used.

## 3. Results

### 3.1. Physical Analyses

**Figure 1:**
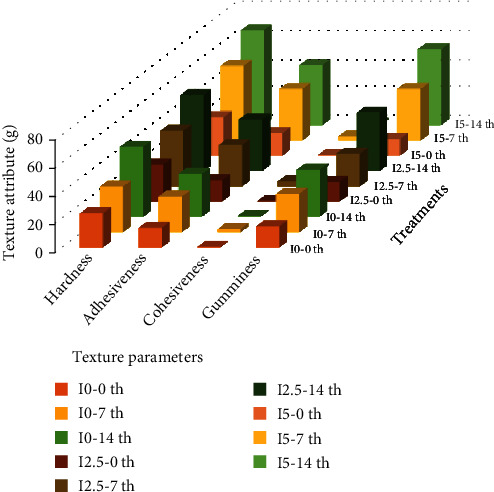
Textural parameters of synbiotic yogurt. I0-0th: inulin 0% -0th day; I0-7th: inulin 0% -7th day; I0-14th: inulin 0% -14th day; I2.5-0th: inulin 2.5% -0th day; I2.5-7th: inulin 2.5% -7th day; I2.5-14th: inulin 2.5% -14th day; I5-0th: inulin 5% -0th day; I5-7th: inulin 5% -7th day; I5-14th: inulin 5% -14th day.

The physical properties of the yogurt samples were determined on the different days of storage and are presented in Table 1 and Figure 1.

The textural parameters showed marked changes with the addition of the different concentrations of inulin in comparison with the control. Adding 2.5% of inulin reduced the hardness, probably because of the fermentation of the available sugars. However, the results showed that adding 5% of inulin increased the hardness. Inulin content, probiotic, and storage time had (*p* < 0.05) increasing effects on WHC. These factors reduced the syneresis and pH of the yogurt samples. As mentioned in other studies, prebiotics and storage time had significant effects (*p* < 0.05) on the viscosity and fat content of yogurt [27]. Changes in the concentration of inulin could improve the viscosity of the product (Table 1).

### 3.2. Sensory Evaluation

**Figure 2:**
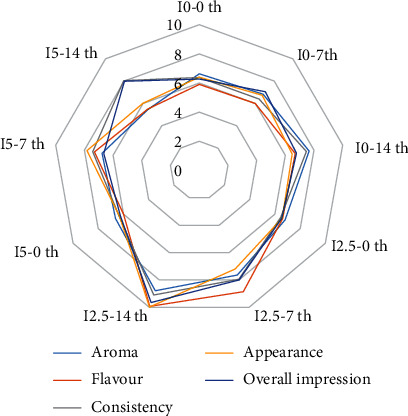
Effects on the sensory attributes of synbiotic yogurt. I0-0th: inulin 0% -0th day; I0-7th: inulin 0% -7th day; I0-14th: inulin 0% -14th day; I2.5-0th: inulin 2.5% -0th day; I2.5-7th: inulin 2.5% -7th day; I2.5-14th: inulin 2.5% -14th day; I5-0th: inulin 5% -0th day; I5-7th: inulin 5% -7th day; I5-14th: inulin 5% -14th day.

Figure 2 shows the significant effect of inulin (*p* < 0.05) on the linear terms in all the sensory properties where the most marked effects were observed on flavor and overall acceptability. As the percentage of inulin increased, the scores improved, but when it reached 5%, the different parameters received low scores (Figure 2), due to the changes in texture and flavor. The aforementioned parameters were evaluated during 2 weeks. The highest percentage of inulin in the yogurt formulation had a positive effect on the flavor.

### 3.3. Chemical Determination

As shown in Table 2, the total soluble solid content of the synbiotic yogurt was significantly (*p* < 0.05) higher than that of the probiotic and control ones on the first day, which will be elaborated in the Discussion.

### 3.4. Antioxidant Activity

**Figure 3:**
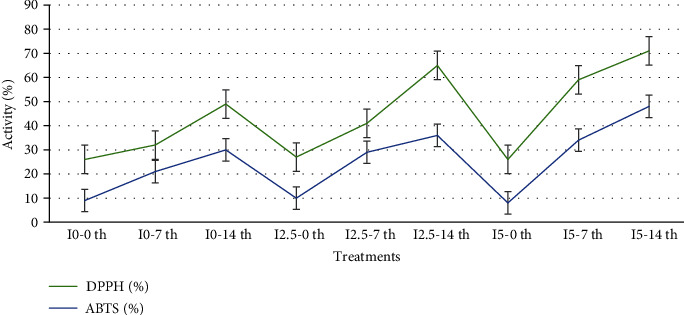
DPPH and ABTS free radical scavenging activity of synbiotic yogurt. I0-0th: inulin 0% -0th day; I0-7th: inulin 0% -7th day; I0-14th: inulin 0% -14th day; I2.5-0th: inulin 2.5% -0th day; I2.5-7th: inulin 2.5% -7th day; I2.5-14th: inulin 2.5% -14th day; I5-0th: inulin 5% -0th day; I5-7th: inulin 5% -7th day; I5-14th: inulin 5% -14th day.

ABTS and DPPH free radical scavenging activity was remarkably high in the samples with the different levels of inulin than in the control. The amount obtained for the sample containing 5% inulin on the 14th day was close to the standard antioxidant, ascorbic acid (69.18 ± 1.00%), and BHA (89.16 ± 2.00%). The antioxidant activities of the different samples are shown in Figure 3.

### 3.5. Microbial Analysis

The number of the probiotics was approximately 6 log CFU.g^−1^ during storage. The probiotic yogurt was significantly more acceptable than the control. The probiotics did not negatively affect the quality of the product, instead increasing its functional, sensory, and qualitative properties. The results also showed that yogurt was an efficient food matrix to maintain the durability of these probiotics during long-term storage [28].

### 3.6. Antimicrobial Assay

**Figure 4:**
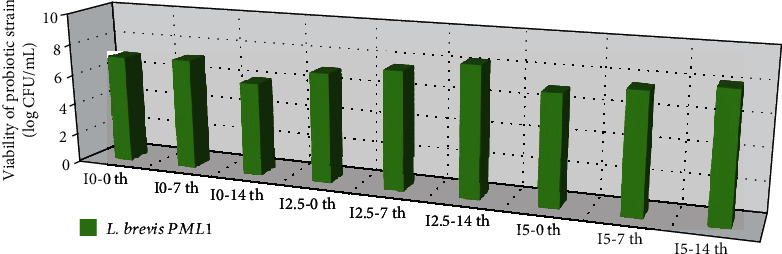
Viability of probiotic (log CFU/g) used in yogurt fermentation during refrigerated storage at 10°C for 14 days. I0-0th: inulin 0% -0th day; I0-7th: inulin 0% -7th day; I0-14th: inulin 0% -14th day; I2.5-0th: inulin 2.5% -0th day; I2.5-7th: inulin 2.5% -7th day; I2.5-14th: inulin 2.5% -14th day; I5-0th: inulin 5% -0th day; I5-7th: inulin 5% -7th day; I5-14th: inulin 5% -14th day.

The results related to the antipathogenic effects of the synbiotic yogurt containing the probiotic strain, *L. brevis* PML1, are summarized in Table 3 and Figure 4. The results showed that the yogurt fermented using *L. brevis* effectively prevented the growth of *S. typhimurium* and *E. coli*. The highest rate of reduction was related to *S. typhimurium*, which occurred on the tenth day in the presence of 5% inulin. By increasing the percentage of inulin, a decrease was observed in the number of the pathogenic bacteria.

## 4. Discussion

The use of inulin caused water to bind with the nonintegrated protein network and ultimately increased the viscosity and hardness of the yogurt samples [29]. Eventually, the accumulation of the casein micelles became stronger. The highest adhesiveness was observed in the sample with 2.5% inulin. The results also showed significant differences in the adhesiveness of the samples and the control. The use of inulin improved the gumminess; nevertheless, it followed no specific trend. Inulin played the roles of a solid network of protein-polysaccharide in the synbiotic yogurt that could improve viscosity and develop a better texture [30]. This result is similar to those of other studies. The increase in acidity during the yogurt storage was due to the activity of the bacteria during fermentation, which converted carbohydrates into lactic acid, CO_2_, and formic acid. During the long-term storage of the yogurt (14 days), syneresis was observed as a major problem, and the separation of the released water from the solid network disrupted the texture and reduced the mouth-feel, resulting in the overall acceptability of the product. Addition of inulin reduced the syneresis of the product, and its amount was inversely correlated with the inulin concentration, so that the lowest amount of syneresis was obtained in the presence of 5% inulin [27]. This happened due to the WHC of inulin, which could also be effective in the long-term storage. The acidity and viscosity of the synbiotic yogurt were improved with an increase in the inulin percentage and storage time [31].

Significantly, taste is an important attribute that plays a key role in the overall acceptability of a product. The effect of inulin on aroma may be due to its ability to hold water [32]. Ehsani et al. reported when prebiotics were added to product at low concentrations, it would not have much effect on the rheological and textural properties as well as the sensory quality of the product, due to their neutral or slightly sweet taste [33]. The viscosity created in the samples could be due to the Maillard reaction in which inulin was the substance that had the greatest effect on the development of the sweet taste. It should be noted that the length of the inulin chain is a critical factor, so that short-chain inulin has the same functional properties as sugar syrup or glucose, while the long-chain inulin has widely been used to replace fat in dairy and fermented products. Several reports have also examined the replacement of sugar and fat with different types of inulin in food products. Sarwar et al. showed that adding inulin to nonfat dairy desserts significantly increased their sweetness and viscosity, compared with the inulin-free samples [34]. Anand et al. reported the good performance of short-chain inulin as a sugar substitute in low-sugar cakes and long-chain inulin as a fat substitute in low-fat cakes. Similar results have been reported in some studies conducted on yogurt [35]. Nooshkam et al. reported that inulin chain length had little effect on the fat-free yogurt color; however, the medium-chain and long-chain inulin gave the yogurt better sensory properties [23]. Kip et al. observed that medium length inulin could be used to improve the creamy feel of low-fat yogurt, while Mazloomi et al. found out that adding too much inulin to yogurt had negative effects on some of the physical and chemical properties of the yogurt [21]. Anand et al. reported that total soluble solids were significantly increased in the food purees containing prebiotic additions (inulin and fructooligosaccharides) compared with the controls [35].

Our results indicated that as the storage time increased, the nutrient content of the samples stored at 4°C decreased gradually. These results are in agreement with those obtained by Ehsani et al. who showed the same trend in the nutrients content of fermented pomegranate juice. The effect of the prebiotic was probably due to the enhanced activity of the *α*-galactosidase produced by the probiotics, which caused the oligosaccharides hydrolysis also in addition to the consumption of simple sugars and proteins. Moreover, inulin slightly increased the solid content of the synbiotic yogurt, while the protein contents of all the samples were not significantly different (*p* < 0.05) before storage [36]. Due to the chemical changes in the yogurt samples during storage, although the addition of inulin did not significantly influence the solid content, the fat and protein contents were significantly reduced in the next stage of storage at low temperatures (*p* < 0.05) [33]. A similar reduction in yogurt fat content has been previously reported by Mazloumi et al. [21].

Elimination of radical oxygen scavengers (ROS) is necessary to maintain normal biological functions for human health. Excessively accumulated ROS in the body causes damage to some important macromolecules such as lipids, proteins, and various nucleic acids, which may cause some diseases such as cancer [37]. Several different bacteria, with the use of appropriate substrates and production of defense compounds, show nonenzymatic immune mechanisms such as reducing the strength and ability of chelating agents to prevent excessive oxidative stress. At the same time, its antioxidant properties prevent the formation of radical cations and peroxidation [38].

This study emphasized that *L. brevis* accompanied by inulin could have many effects on harmful foodborne pathogens to increase the safety and shelf-life of fermented yogurt. Fermented products such as yogurt can be proper media for the survival and reproduction of probiotic strains, so they would be able to exert their health effects. Our results indicated that storage time had significant effects (*p* < 0.05) on the viability of *L. brevis* in the product [39].

The viability of probiotic bacteria with the addition of another polymer such as *β*-glucan has been investigated by Salami et al. and Friday et al. With an increase in inulin concentration, minerals such as nitrogen and the carbon sources required for the bacteria were also elevated, and the viability rate of the probiotic bacteria rose significantly (*p* < 0.05). Therefore, more probiotics could survive in the final product. These results are in contradiction to those obtained by Sarwar et al. in the case of cow yogurt [34]. Ladjevardi et al. showed that prebiotic ingredients were more effective than inulin on bacterial viability, yogurt texture, and protein-polysaccharide network formation. This can be shown by the consumption of nutrients by microorganisms. It should be noted that the production of some organic acids by probiotics makes the conditions more unfavorable for the growth of bacteria. The results showed that the number of the remaining bacteria after incubation in the simulated intestinal juice was larger in the synbiotic yogurt than in the control. Beneficial microorganisms should be kept viable in fermented milk products such as yogurt before consumption. According to many scientific studies, inulin, as a fermentation substrate, has been reported to be beneficial for the growth of probiotics in the production of fermented milk products [40]. In general, the total number of LAB in yogurt was more than at least 10^7^ living microorganisms per gram. Additionally, their viability was raised during the 14 days of refrigeration, which indicates that our yogurt formulations provided a suitable food matrix for the growth and viability of these bacteria [12].

In general, gastrointestinal pathogens may produce toxins that block epithelial cell function and the body metabolic repression, causing biological diseases including colon cancer and different syndromes [7]. Previous studies have shown that the overgrowth of pathogenic bacteria significantly reduces the role of health-promoting bacteria in the innate inflammation and infection of the gastrointestinal tract. ROS, on the other hand, which mainly occurs during oxidative metabolism, can lower the risk of diverse gastrointestinal diseases. Fortunately, several studies have shown that the foods containing probiotics as live microbial dietary supplements have beneficial effects on the prevention and treatment of various gastrointestinal disorders. The antimicrobials produced by probiotics may not only reduce the number of living pathogenic cells but also affect the bacterial metabolism and toxin production [41]. Important mechanisms have also been developed to promote intestinal homeostasis, stabilize or maintain gastrointestinal barrier function, and suppress ROS-induced oxidative stress and carcinogenic enzymatic activities [8]. Prebiotics that are available in significant parts in several foods can change the colonic microbiota to a healthy composition by producing beneficial effects in the host body. Inulin can have a protective effect on probiotic cultures, including increasing the viability and activity of the probiotic cultures during storage. This effect is used to describe inulin as a suitable substrate for metabolism and fermentation. The use of inulin also causes a rise in the growth of bacteria and the secretion of nutrients such as amino acids. Inulin can also protect the bacterial cells from environmental damage by raising their viability [18].

Lactic acid and acetic acid were produced in the synbiotic yogurt through the hetero-fermentation of *L. brevis*. As a general rule, the antibacterial activity of synbiotic yogurt has more influence on Gram-negative bacteria rather than the Gram-negative ones. During fermentation, LAB strains produce only lactic acid, while the non-hetero-fermented LAB strains produce various antimicrobials such as lactic acid, acetic acid, alcohol, carbon dioxide, formic acid, acetone, acetaldehyde, and diacetyl [38].

## 5. Conclusion

A novel synbiotic yogurt with desirable quality was developed as an effective carrier for the delivery of a probiotic exerting its beneficial health effects. The important result in this study was that the textural properties were improved at high percentages of inulin during storage, but syneresis and fat content increased when small amounts of inulin were used. pH changes were in accordance with the syneresis changes. WHC and bacterial changes were similar to those of the inulin concentration and storage time. In addition, the antioxidant and antimicrobial effects were greater in the samples containing higher inulin contents during the 14 days of storage, and the results showed that the synbiotic yogurt could be a good inhibitor against foodborne pathogens. The results also showed the synbiotic yogurt consequently improved the gastrointestinal functions and the immune system.

## Figures and Tables

**Table 1:** Physical properties of samples under different treatments.

Treatment	pH	Acidity (%)	Syneresis (*v*/*w*)	Viscosity (cps)	WHC (%)
I0^∗^-0th^∗∗^	4.41 ± 0.01	0.16 ± 0.04	10.5 ± 0.2	1115.1 ± 2.2	10.25 ± 0.21
I0-7th^∗∗∗^	4.25 ± 0.04	0.26 ± 0.07	11.8 ± 0.7	1215.3 ± 4.3	11.24 ± 0.42
I0-14th^∗∗∗∗^	4.18 ± 0.02	0.32 ± 0.07	12.5 ± 0.4	1295.2 ± 2.5	13.40 ± 0.63
I2.5-0th	3.82 ± 0.03	0.18 ± 0.01	11.4 ± 0.5	1065.4 ± 4.1	12.25 ± 0.21
I2.5-7th	3.73 ± 0.03	0.36 ± 0.05	12.6 ± 0.6	1313.7 ± 5.7	12.44 ± 0.30
I2.5-14th	3.71 ± 0.02	0.47 ± 0.06	14.9 ± 0.3	1425.9 ± 6.3	13.21 ± 0.24
I5-0th	3.54 ± 0.05	0.18 ± 0.08	11.3 ± 0.4	1105.3 ± 7.3	15.25 ± 0.15
I5-7th	3.44 ± 0.06	0.35 ± 0.03	14.7 ± 0.1	1462.1 ± 7.6	15.42 ± 0.21
I5-14th	3.12 ± 0.05	0.58 ± 0.02	16.2 ± 0.2	1636.6 ± 5.0	16.36 ± 0.12

^∗^I represent the different levels of inulin (0, 2.5, and 5); ^∗∗^, ^∗∗∗^, and ^∗∗∗∗^ represent the storage time (day). I0-0th: inulin 0% -0th day; I0-7th: inulin 0% -7th day; I0-14th: inulin 0% -14th day; I2.5-0th: inulin 2.5% -0th day; I2.5-7th: inulin 2.5% -7th day; I2.5-14th: inulin 2.5% -14th day; I5-0th: inulin 5% -0th day; I5-7th: inulin 5% -7th day; I5-14th: inulin 5% -14th day.

**Table 2:** Chemical properties of synbiotic yogurt under specified conditions.

Treatment	Total solid	Protein	Fat	Color parameters		
				*L* ^∗^	*a* ^∗^	*b* ^∗^
I0-0th	17.48 ± 0.15	3.89 ± 0.01	3.41 ± 0.14	68.6 ± 0.1	4.1 ± 0.4	10.1 ± 0.2
I0-7th	18.38 ± 0.56	3.77 ± 0.04	3.38 ± 0.21	66.7 ± 0.4	4.7 ± 0.4	11.3 ± 0.3
I0-14th	21.13 ± 0.43	3.19 ± 0.02	3.21 ± 0.32	65.3 ± 0.5	5.1 ± 0.2	12.8 ± 0.5
I2.5-0th	16.98 ± 0.21	3.86 ± 0.02	3.45 ± 0.43	67.4 ± 0.2	4.2 ± 0.3	11.5 ± 0.7
I2.5-7th	19.36 ± 0.13	3.79 ± 0.03	3.35 ± 0.35	68.5 ± 0.3	4.1 ± 0.1	11.6 ± 0.5
I2.5-14th	23.43 ± 0.63	3.62 ± 0.03	3.22 ± 0.22	69.2 ± 0.6	4.5 ± 0.2	11.7 ± 0.3
I5-0th	17.77 ± 0.15	3.89 ± 0.04	3.51 ± 0.56	66.3 ± 0.3	4.2 ± 0.1	11.8 ± 0.6
I5-7th	20.16 ± 0.22	3.59 ± 0.05	3.25 ± 0.61	64.7 ± 0.8	4.6 ± 0.3	13.3 ± 0.2
I5-14th	25.18 ± 0.23	3.13 ± 0.05	3.01 ± 0.13	63.9 ± 0.4	4.3 ± 0.2	14.6 ± 0.3

I0-0th: inulin 0% -0th day; I0-7th: inulin 0% -7th day; I0-14th: inulin 0% -14th day; I2.5-0th: inulin 2.5% -0th day; I2.5-7th: inulin 2.5% -7th day; I2.5-14th: inulin 2.5% -14th day; I5-0th: inulin 5% -0th day; I5-7th: inulin 5% -7th day; I5-14th: inulin 5% -14th day.

**Table 3:** Effect of inulin concentrations on antibacterial activity (% reduction) of *L. brevis* PML1 used as probiotic yogurt starter.

Synbiotic yogurt (% inulin)	Inhibition (%)							
	*S. aureus*		*L. innocua*		*E. coli*		*S. typhimurium*	
	5	10	5	10	5	10	5	10
0	8.2 ± 0.7	6.4 ± 0.8	7.2 ± 1.3	6.8 ± 0.7	11.8 ± 0.3	9.5 ± 0.9	20.6 ± 1.3	19.3 ± 0.8
2.5	11.9 ± 0.2	8.9 ± 0.5	8.6 ± 1.9	9.5 ± 1.4	10.2 ± 0.7	9.8 ± 0.1	25.1 ± 1.5	26.7 ± 0.6
5	14.2 ± 1.2	12.7 ± 0.6	9.3 ± 0.8	10.9 ± 2.1	16.6 ± 0.6	14.5 ± 0.5	28.7 ± 2.2	26.9 ± 1.6

## Data Availability

I want to inform you that all data generated or analyzed during this study are included in this published article, and also, all data that support the findings of this study are openly available.
